# Phytoestrogen *α*-Zearalanol Improves Memory Impairment and Hippocampal Neurogenesis in Ovariectomized Mice

**DOI:** 10.1155/2014/862019

**Published:** 2014-07-21

**Authors:** Yilong Dong, Aimei Jiang, Hongju Yang, Huicheng Chen, Yanmei Wang

**Affiliations:** ^1^School of Medicine, Yunnan University, Kunming, Yunnan 650091, China; ^2^The First Affiliated Hospital of Kunming Medical University, Kunming, Yunnan 650031, China

## Abstract

Estrogen is known to provide robust protection of memory in postmenopausal women, but the fact that estrogen may increase the incidence of uterine and breast tumors has undoubtedly limited the clinical use of estrogen. In the present study, the effect of *α*-zearalanol (*α*-ZAL), a plant-derived phytoestrogen with low side-effect on uterine and breast, on memory has been evaluated in ovariectomized (OVX) mice when using 17*β*-estradiol (17*β*-E2) as an estrogen positive control. Our findings demonstrated that OVX resulted in impaired spatial learning and memory and reduced numbers of newborn neurons in the dentate gyrus of the hippocampus, while 17*β*-E2 or *α*-ZAL treatment significantly improved memory performance and restored hippocampal neurogenesis. We also found the reduction of brain derived neurotrophic factor (BDNF) and TrkB expression in OVX mice, which were ameliorated by 17*β*-E2 or *α*-ZAL supplementation. These results indicated that *α*-ZAL may improve memory impairments induced by OVX and modulate the expression of BDNF-TrkB benefit to neurogenesis which may be involved in the memory protection from *α*-ZAL, in a manner similar to that of 17*β*-E2. The present findings suggested that *α*-ZAL may be a plausible substitute of 17*β*-E2 in improving memory in postmenopausal women.

## 1. Introduction

It is widely acknowledged that adult neurogenesis is a continuous physiological process throughout life in several species, including humans [1]. In mammals, neurogenesis occurs in two areas of the central nervous system (CNS): the subgranular zone of the dentate gyrus (DG) of the hippocampus and the subventricular zone (SVZ) of the lateral ventricles [2]. Rodent studies have shown adult DG neurogenesis is important in mediating hippocampal-dependent learning and memory, and the reduced in neurogenesis is a causative factor of memory impairments [3–6], therefore, enhance neurogenesis can lead to memory improvement. In relation to this, increase expression of neurotrophic factors such as brain derived neurotrophic factor (BDNF), shall be contribute to alleviate memory loss since BDNF is clearly involved in the beneficial effects of newborn neurons generation and survival [7].

Beyond the classic role in reproduction, estrogen has been shown to have multifaceted neuroprotection, such as facilitate axonal sprouting, improve synaptic transmission, and enhance neurotrophic factors [8]. These beneficial effects have made the use of estrogen replacement therapy as an effective method to improve memory decline in postmenopausal women who burden a higher risk of cognitive impairments than premenopausal women [9]. However, the clinical application of estrogen is still in controversy due to the fact that estrogen may increase the risk of developing breast and endometrial cancer in women [10]. In our published data, we have reported that *α*-zearalanol (*α*-ZAL), a plant-derived phytoestrogen, may effectively maintain estrogen level in ovariectomized rats and alleviated neuron loss induced by estrogen deficiency, but the side effect on uterine is less than estrogen [11]. Also, there were evidences that showed that the carcinogenic effect of *α*-ZAL on breast tissue is significantly less than estrogen [12].These findings collectively indicated that *α*-ZAL may be used as a safe alternative for estrogen. However, it remains elusive whether the *α*-ZAL exerts the similar power on memory as estrogen. Therefore, in the present study, we investigated the effect of *α*-ZAL on memory in ovariectomized mice when 17*β*-estradiol (17*β*-E2) has been used as an estrogen positive control, followed by measurement of hippocampal neurogenesis and neurotrophic factors in order to determine the potential mechanism involved in *α*-ZAL.

## 2. Materials and Methods

### 2.1. Materials

*α*-ZAL was a gift from Professor Shunling Dai at Perking Union Medical College. 17*β*-E2 and 5′-bromo-2′-deoxyuridine (BrdU) were purchased from Sigma-Aldrich (Saint Louis, MO, USA). The protease inhibitor mixture and BCA Protein Assay Kit were purchased from Pierce Biotechnology (Rockford, IL, USA). BrdU antibody was purchased from Millipore (Billerica, MA, USA). BDNF, TrkB, and p75NTR antibody were purchased from Abcam (Abcam, England); *β*-actin antibody was purchased from Santa Cruz Biotechnology (Santa Cruz, CA, USA). The avidin-biotin-peroxidase and DAB kits for immunohistochemical detection were obtained from Zhongshan Goldenbridge Biotechnology (Beijing, China). All other chemicals used were of the highest grade commercially available.

### 2.2. Animals and Treatment

Female C57/BL6 mice (8-week-old) were purchased from Weitong Lihua Experimentary Animal Central (Beijing, China). Animals were housed in groups of five on a 12-hour light/dark schedule and had free access to food and water at all times. Animal treatment and maintenance were carried out in accordance the guidelines established by the National Institutes of Health for the care and use of laboratory animals and were approved by the Animal Care Committee of the Yunnan University.

After 5 days of habituation, animals were anesthetized with ketamine (100 mg/kg) and xylazine (20 mg/kg) and bilateral ovariectomy (OVX) or sham-operation was performed. Following surgery, the mice were allowed to recover for 14 days and then assigned to one of the following four groups (*n* = 10 in each group): control group: sham-operation + olive oil; OVX group: OVX + olive oil; 17*β*-E2 group: OVX + 17*β*-E2; *α*-ZAL group: OVX + *α*-ZAL. Mice from 17*β*-E2 group and *α*-ZAL group received intraperitoneal injection (i.p) 17*β*-E2 (0.5 mg/kg) or *α*-ZAL (0.5 mg/kg), respectively. 17*β*-E2 and *α*-ZAL were dissolved in olive oil. Mice in control group and OVX group received an equal volume of the olive oil. The intraperitoneal injections were given every three days. Treatment continued for 8 weeks. The above animal treatment procedure was carried out in accordance with our published part [11].

### 2.3. Morris Water Maze

At the 52nd day after treatment, the Morris water maze task was used to evaluate spatial learning and memory. This process of Morris water maze consisted of 4-day learning and memory training and a probe trial on day 5 as described [13]. Animals were trained in a circular pool (100 cm in diameter) located in a lit room with visual cues. A black escape platform (9.5 cm in diameter) was submerged 1.0 cm below the surface of the pool water, which was maintained at 21 ± 1°C, and mixed with milk powder to obscure the platform. In the training trail, the mice were pretrained to find the hidden platform and the location of the platform remained in the center of northwest quadrant throughout the 4-day training period. Three trials per day were conducted. Each mouse was placed by the tail into the water immediately, facing the perimeter, in a fixed position opposite the hidden platform quadrant and given 60 s to find the hidden platform in each trial. The time required to escape onto the hidden platform (escape latency) was recorded. If the mouse did not find the platform within this time, then the experimenter led the mouse to the platform where it sat for 10 s, and the escape latency was recorded as 60 s. The probe trial was made by removing the platform and allowing each mouse to swim freely for 60 s inside the pool. The time spent in the northwest quadrant (explore time, where the platform was originally placed), the escape latency (time to reach the originally platform position), and the swimming speed were recorded with a computerized video system (Beijing Sunny Instruments Co. Ltd., China).

### 2.4. BrdU Injections

To investigate the neurogenesis in hippocampus, the thymidine analog BrdU was injected intraperitoneally (50 mg/kg per injection, every 8 h for 24 h) at the last day of treatment.

### 2.5. Tissue Preparation

Mice were sacrificed 24 h after the last behavioral test, 6 animals in each group will be deeply anesthetized by isoflurane inhalation and decapitation, and the brain was removed and dissected on ice. Hippocampus was removed and stored at −80°C for western blotting analysis. The remaining 4 animals will be perfused with phosphate-buffered saline (PBS) and 4% paraformaldehyde under deeply anaesthetic condition; brains were removed and postfixed in 10% sucrose/4% paraformaldehyde for 8 h at 4°C and then placed in 20% sucrose/PBS at 4°C overnight. Using a cryostat microtome, 25 *μ*m frozen sections were cut for BrdU experiment.

### 2.6. BrdU Immunohistochemisty

Sections were pretreated with 2 M HCl at 37°C for 30 min and rinsed in 0.1 M boric acid (pH 8.5) at room temperature for 10 min. Sections were then incubated in 0.3% H_2_O_2_ for 15 min, followed by incubation for 2 h in blocking solution at room temperature, and sequentially incubated with anti-BrdU (1 : 100 dilution) at 4°C overnight. After washing with PBS, the sections were incubated with biotinylated goat anti-mouse IgG for 1 h, followed by incubation for 1 h in an avidin-biotin-peroxidase complex solution. Visualization was done by 3,3-diaminobenzidine (DAB). Sections were examined with a Leica microscope. The number of BrdU immunoreactive cells in the dentate gyrus was counted in six coronal hippocampal sections per animal.

### 2.7. Western Blot

Hippocampal tissues were homogenized in lysis buffer (150 mM NaCl, 1% NP-40, 0.5% sodium deoxycholate, 0.1% SDS, 5 mM ethylenediaminetetraacetic acid, 50 mM Tris-Hcl, pH 8.0) supplemented with protease inhibitor cocktail and then centrifuged at 12,000 rpm for 15 min at 4°C. Afterward, the supernatant was collected and protein concentration was estimated by BCA Protein Assay Kit. 50 *μ*g protein has been loaded onto a 10% gradient polyacrylamide gel, electrophoretically transferred to polyvinylidene difluoride membrane; after being incubated for 2 h at room temperature with blocking buffer, membranes were then incubated with primary antibodies: anti-BDNF (1 : 1000), anti-TrkB (1 : 1000), anti-p75NTR (1 : 1000), and anti-*β*-actin (1 : 2000) for 12 h at 4°C. After washing, the membranes were incubated with goatradish peroxidase-conjugated secondary antibody (1 : 5000) for 2 h at room temperature. The blots were developed using an enhanced chemiluminescent assay. Scanned images of the developed blots were quantified using densitometry functions in Image-Pro Express 4.0.

### 2.8. Statistical Analysis

Data were expressed as mean ± SD. Differences between mean values were analyzed using one-way ANOVA followed by post-hoc tests. Statistical significance was set at *P* ≤ 0.05.

## 3. Results

### 3.1. Effects of *α*-ZAL on Spatial Learning and Memory

**Figure 1:**
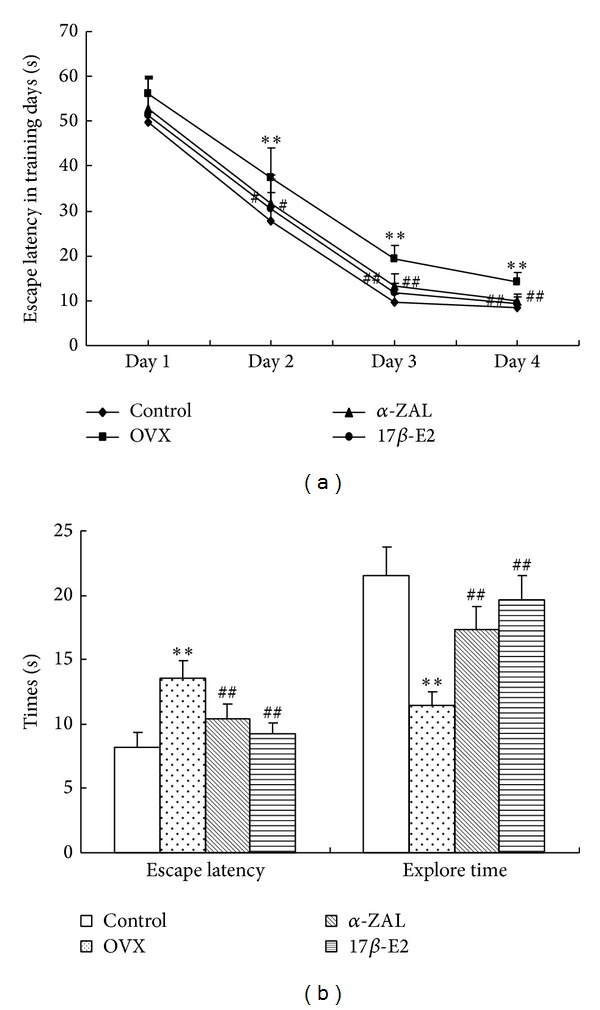
(a) The escape latencies of mice to find the submerged platform time during the Morris water maze at the training trials session. (b) Escape latencies and explore time at the probe test session. 17*β*-E2 or *α*-ZAL treatment significantly improves the decline of learning and memory induced by OVX. **P < 0.01 versus control group; ^#^ *P* < 0.05 and ^##^ *P* < 0.01 versus OVX group. (*n* = 10 in each group).

As shown in Figure 1(a), although all the mice showed a progressive decrease in escape latencies at the training phase, significantly longer latencies were observed in OVX mice than in the control mice from training day 2 to day 4. These results suggested that control mice were better able to learn this task than OVX mice. Also, significantly shorter latencies were observed in 17*β*-E2 or *α*-ZAL treated mice compared to OVX mice at the same training days, which indicated that 17*β*-E2 or *α*-ZAL supplementation is a benefit to learning function. Moreover, the mice's memory function was evaluated in probe trials. As shown in Figure 1(b), OVX mice showed significant increase in escape latency but decrease in the time spent in target quadrant as compared to control animal during the probe test. Treatments with 17*β*-E2 or *α*-ZAL improved spatial memory impairment as indicated by the significant shorten latency time and lengthen explore time as compared to OVX mice. Training and probe trials study also revealed that 17*β*-E2 and *α*-ZAL treated mice showed no significant different in latency time and/or explore time. Furthermore, there were no significant differences in the speeds between different groups (data were not shown); therefore, the effects of motivational (swimming speed) factor on animal's learning and memory performance could be excluded.

### 3.2. Effects of *α*-ZAL on the Hippocampal Neurogenesis

**Figure 2:**
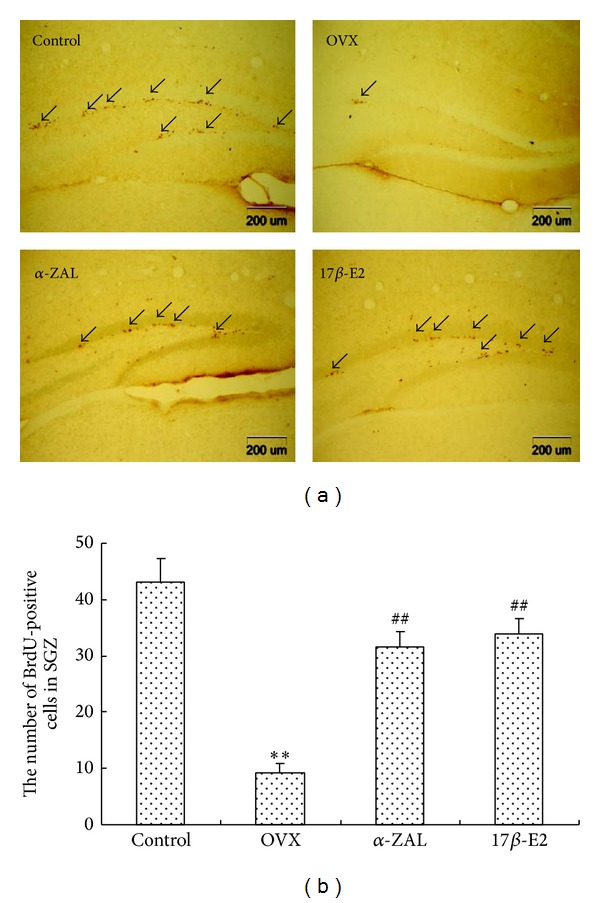
(a) Photomicrograph of 5-bromo-2-deoxyuridine (BrdU) immunopositive cells (new born neurons) in DG in the hippocampus of control and OVX mice with 17*β*-E2 or *α*-ZAL treatment. Fewer BrdU-labeled cells were observed in OVX mice compared to control. 17*β*-E2 and *α*-ZAL-treated mice expressed higher BrdU^+^ cells compared to OVX animal. Magnification ×40. (b) Quantification of BrdU immunolabeling cells in DG. *n* = 4 in each group and the result indicated at least three independent experiments in each animal. **P < 0.01 versus control group; ^##^ *P* < 0.01 versus OVX group.

As shown in Figure 2, newly generated BrdU-labeled cells were observed in the dentate gyrus (DG) of the hippocampal. The BrdU-positive cells were irregular in shape and many proliferating cells were found in clusters. Quantitative analysis of BrdU-labeled cell within the DG revealed a sharp decrease in OVX mice comparison with controls, but these changes were attenuated by 17*β*-E2 or *α*-ZAL supplementation.

### 3.3. Effects of *α*-ZAL on BDNF, TrkB, and p75NTR Protein Expression

**Figure 3:**
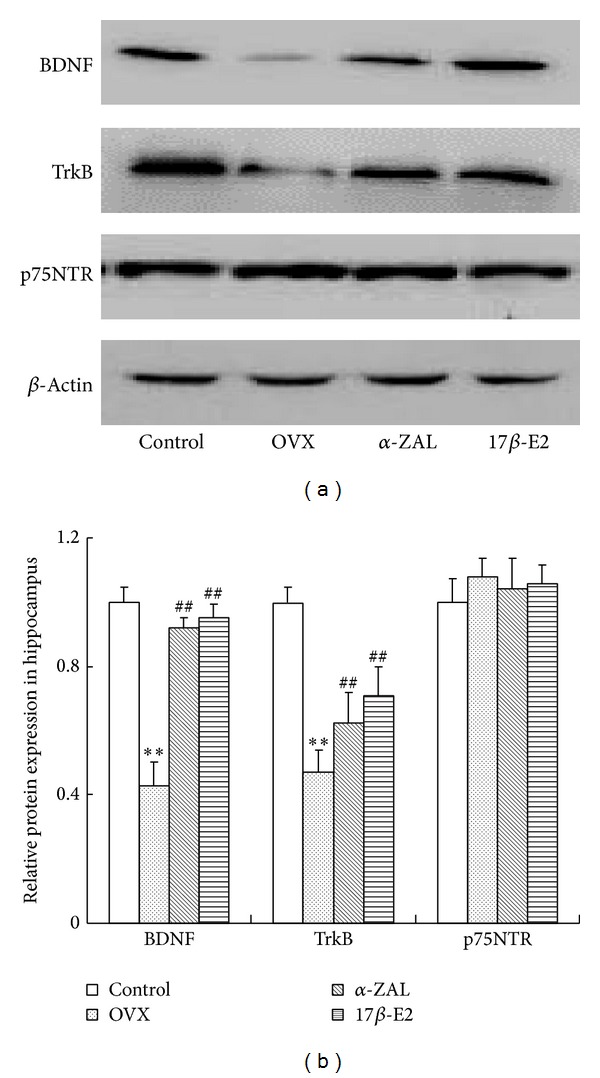
(a) Western blotting analysis the expression of BDNF, TrkB, and p75NTR in hippocampus. (b) Quantitative analysis of protein levels by densitometry. The data from western blot were normalized by taking the value of control group as 1. **P < 0.01 versus control group; ^##^ *P* < 0.01 versus OVX group.

As shown in Figure 3, western blot analysis revealed a significant decrease in BDNF protein expressions in OVX animals in comparison with those of control animals, while *α*-ZAL supplementation effectively ameliorated this decline, as did 17*β*-E2. Corresponding to BDNF result, OVX significantly downregulated TrkB, and this reduction induced by OVX can be attenuated by *α*-ZAL or 17*β*-E2 administration. In contrast, although there was a slight elevation in p75NTR expression in OVX mice, there was no statistics difference between different groups.

## 4. Discussion

Estrogen replacement therapy has proven to be effective in preventing memory and learning deficiencies in postmenopausal women [9]. However, the increased risk of breast and endometrial cancer produced by estrogen supplementation has undoubtedly limited the use of estrogen [10]. Therefore, the search for a safe and effective estrogen substitute gets more attention. In the present study, the effect of *α*-ZAL, a low side-effect phytoestrogen, on cognitive function has been evaluated. Our results demonstrated that spatial learning and memory were improved by *α*-ZAL supplementation; moreover, *α*-ZAL also restored the decrease of hippocampal neurogenesis and neurotrophic factor level induced by estrogen deficiency, just like 17*β*-E2. The findings indicated that the effect involved in *α*-ZAL mediated memory improvement in a manner similar to that of 17*β*-E2.

Numerous experiments have directly examined the effects of neurogenesis on memory. For example, Thuret and colleagues found that the spatial learning and recognition learning declined in MRL/MpJ mice (an animal with low levels of adult neurogenesis) compared to normal mice [14]. Similarly, Deng et al. also demonstrated that the mice with a reduced population of newborn neurons were deficient in forming long-term spatial memory and displayed impaired performance in behavioral tasks [3]. In this study, our Morris water maze results from training phase suggested that the mice in control group were better able to learn the water maze task; however, the OVX mice exhibited a decline in learning ability. Moreover, our present data from probe trials also have shown that OVX mice exhibited longer latency time but shorter explore time in the water-maze compared to control animal, indicating impairment in their spatial working memory. Consistent with these findings, we found the number of newly generating BrdU-labeled cells in the DG decreased sharply in OVX mice. Simultaneously, the present data also have shown that the reduction of hippocampal neurogenesis and learning and memory deficits were reversed by 17*β*-E2 or *α*-ZAL administration. These findings corroborated that *α*-ZAL may provide major benefit for hippocampal neurogenesis and subsequently resulted in cognitive improvement, as did 17*β*-E2.

As a positive modulator of physiological neurogenesis, several lines of evidence indicate that estrogen play a role in hippocampal neurogenesis [15, 16]. Although the mechanism of estrogen on neurogenesis needs more exploring, there are studies that have uncovered BDNF involved in this process [17]. BDNF is a key neurotrophin that is highly expressed in the hippocampus and plays critical roles in the nervous system in neuronal development, differentiation, and synaptic plasticity. The biological effects of BDNF on cells are through corresponding receptors classical as high-affinity receptors tyrosine kinase (Trk) B, or through the common low-affinity receptor p75NTR receptor. When binding with TrkB receptor, the activation of several intracellular pathways occurs, including the mitogen-activated protein kinase (MAPK) pathway, and the phosphoinositide 3-kinase (PI3 K)/Akt/Bcl2 pathway which promotes newborn neuron survival and differentiation [18]. Indeed, the TrkB receptor is present on newborn cells and its deficiency impairs adult neurogenesis that have been defined [19, 20]. Unlike TrkB, p75NTR shows contradictory effects depending on the cells presence or absence of Trk receptors. p75NTR induces the neurotrophins mediated survival in neuronal cells expressing TrkB; when there is reduction of TrkB activation or low Trk/p75NTR ratio, apoptosis occurs upon neurotrophins binding to p75 [21]. In agreement with Pan et al.'s report [17], the present study demonstrated that the downregulation of BDNF and TrkB induced by OVX has been attenuated by 17*β*-E2 supplementation. Corresponding to the reduction of neurotrophic, numbers of BrdU-positive cells in OVX animals were decreased sharply compared to control, while 17*β*-E2 treatment reversed these changes. Importantly, the same beneficial effects in neurogenesis and neurotrophic factor also have been observed when animals treated with *α*-ZAL. Accordingly, we speculated that *α*-ZAL may share the parallel protective mechanism on neurogenesis with 17*β*-E2. Moreover, our findings also implicated that neuroprotective effects mediated by TrkB signal are predominant when the animals received 17*β*-E2 or *α*-ZAL since there was no significant changes in p75NTR expression in different groups. Although the exact mechanisms by which *α*-ZAL regulate neurotrophin are unknown, estrogen receptor maybe involved in this process since there was evidence that demonstrated that *α*-ZAL own the ability to interact with estrogen receptor [22] and which have been confirmed to support BDNF expression [23].

## 5. Conclusion

In summary, in the current study, we compared the power of 17*β*-E2 and *α*-ZAL on memory deficit induced by low estrogen level and explored that memory protective effect of *α*-ZAL at least in part, attributable to its property improve hippocampal neurogenesis, and BDNF-TrkB play a role in *α*-ZAL mediated new neuron generate, just like as 17*β*-E2. Although there were still some limitations in this work, for example, (1) only the medium concentration of *α*-ZAL has been investigated and (2) BDNF silence or estrogen receptor antagonist has not been used to analyze the precise molecular mechanism of *α*-ZAL on neurogenesis, it seems that *α*-ZAL may be a plausible substitute of estrogen for preventing memory decline in postmenopausal women.

## References

[1] D'Alessio L, Konopka H, López EM (2010). Doublecortin (DCX) immunoreactivity in hippocampus of chronic refractory temporal lobe epilepsy patients with hippocampal sclerosis. *Seizure*.

[2] Gould E, Reeves AJ, Graziano MSA, Gross CG (1999). Neurogenesis in the neocortex of adult primates. *Science*.

[3] Deng W, Saxe MD, Gallina IS, Gage FH (2009). Adult-born hippocampal dentate granule cells undergoing maturation modulate learning and memory in the brain. *Journal of Neuroscience*.

[4] Sahay A, Scobie KN, Hill AS (2011). Increasing adult hippocampal neurogenesis is sufficient to improve pattern separation. *Nature*.

[5] Shors TJ, Miesegaes G, Beylin A, Zhao M, Rydel T, Gould E (2001). Neurogenesis in the adult is involved in the formation of trace memories. *Nature*.

[6] Saxe MD, Battaglia F, Wang J (2006). Ablation of hippocampal neurogenesis impairs contextual fear conditioning and synaptic plasticity in the dentate gyrus. *Proceedings of the National Academy of Sciences of the United States of America*.

[7] Grote HE, Hannan AJ (2007). Regulators of adult neurogenesis in the healthy and diseased brain. *Clinical and Experimental Pharmacology and Physiology*.

[8] Aguirre CC, Baudry M (2009). Progesterone reverses 17*β*-estradiol-mediated neuroprotection and BDNF induction in cultured hippocampal slices. *European Journal of Neuroscience*.

[9] Valen-Sendstad A, Engedal K, Stray-Pedersen B (2010). Effects of hormone therapy on depressive symptoms and cognitive functions in women with alzheimer disease: a 12 month randomized, double-blind, placebo-controlled study of low-dose estradiol and norethisterone. *The American Journal of Geriatric Psychiatry*.

[10] Chlebowski RT, Anderson GL, Gass M (2010). Estrogen plus progestin and breast cancer incidence and mortality in postmenopausal women. *Journal of the American Medical Association*.

[11] Dong Y-L, Yue Y, Liu F-H (2006). Treatment with phytoestrogen *α*-zearalanol might protect neurons of hippocampus in ovariectomized rats. *Endocrine*.

[12] Deng W, Dai S, Zhang Y, Duan J, Wu Y (2010). The effects of *α*-zearalanol and estradiol benzoate on expression of c-myc, c-fos and epidermal growth factor receptor mRNAs in breast tissues implanted into nude mice. *Gynecological Endocrinology*.

[13] Li L, Xiao N, Yang X (2012). A high cholesterol diet ameliorates hippocampus-related cognitive and pathological deficits in ovariectomized mice. *Behavioural Brain Research*.

[14] Thuret S, Toni N, Aigner S, Yeo GW, Gage FH (2009). Hippocampus-dependent learning is associated with adult neurogenesis in MRL/MpJ mice. *Hippocampus*.

[15] McClure RES, Barha CK, Galea LAM (2013). 17*β*-Estradiol, but not estrone, increases the survival and activation of new neurons in the hippocampus in response to spatial memory in adult female rats. *Hormones and Behavior*.

[16] Kordower JH, Chen E, Morrison JH (2010). Long-term gonadal hormone treatment and endogenous neurogenesis in the dentate gyrus of the adult female monkey. *Experimental Neurology*.

[17] Pan M, Li Z, Yeung V, Xu RJ (2010). Dietary supplementation of soy germ phytoestrogens or estradiol improves spatial memory performance and increases gene expression of BDNF, TrkB receptor and synaptic factors in ovariectomized rats. *Nutrition and Metabolism*.

[18] Bath KG, Mandairon N, Jing D (2008). Variant brain-derived neurotrophic factor (Val66Met) alters adult olfactory bulb neurogenesis and spontaneous olfactory discrimination. *Journal of Neuroscience*.

[19] Bergami M, Rimondini R, Santi S, Blum R, Götz M, Canossa M (2008). Deletion of TrkB in adult progenitors alters newborn neuron integration into hippocampal circuits and increases anxiety-like behavior. *Proceedings of the National Academy of Sciences of the United States of America*.

[20] Donovan MH, Yamaguchi M, Eisch AJ (2008). Dynamic expression of TrkB receptor protein on proliferating and maturing cells in the adult mouse dentate gyrus. *Hippocampus*.

[21] Mamidipudi V, Wooten MW (2002). Dual role for p75NTR signaling in survival and cell death: can intracellular mediators provide an explanation?. *Journal of Neuroscience Research*.

[22] Dai S, Duan J, Lu Y (2004). Phytoestrogen *α*-zearalanol inhibits atherogenesis and improves lipid profile in ovariectomized cholesterol-fed rabbits. *Endocrine*.

[23] Spencer-Segal JL, Tsuda MC, Mattei L (2012). Estradiol acts via estrogen receptors alpha and beta on pathways important for synaptic plasticity in the mouse hippocampal formation. *Neuroscience*.

